# Multiple White Matter Volume Reductions in Patients with Panic Disorder: Relationships between Orbitofrontal Gyrus Volume and Symptom Severity and Social Dysfunction

**DOI:** 10.1371/journal.pone.0092862

**Published:** 2014-03-24

**Authors:** Jun Konishi, Takeshi Asami, Fumi Hayano, Asuka Yoshimi, Shunsuke Hayasaka, Hiroshi Fukushima, Thomas J. Whitford, Tomio Inoue, Yoshio Hirayasu

**Affiliations:** 1 Department of Psychiatry, Graduate School of Medicine, Yokohama City University, Yokohama, Japan; 2 School of Psychology, University of New South Wales, Sydney, New South Wales, Australia; 3 Department of Radiology, Graduate School of Medicine, Yokohama City University, Yokohama, Japan; Hangzhou Normal University, China

## Abstract

Numerous brain regions are believed to be involved in the neuropathology of panic disorder (PD) including fronto-limbic regions, thalamus, brain stem, and cerebellum. However, while several previous studies have demonstrated volumetric gray matter reductions in these brain regions, there have been no studies evaluating volumetric white matter changes in the fiber bundles connecting these regions. In addition, although patients with PD typically exhibit social, interpersonal and occupational dysfunction, the neuropathologies underlying these dysfunctions remain unclear. A voxel-based morphometry study was conducted to evaluate differences in regional white matter volume between 40 patients with PD and 40 healthy control subjects (HC). Correlation analyses were performed between the regional white matter volumes and patients' scores on the Panic Disorder Severity Scale (PDSS) and the Global Assessment of Functioning (GAF). Patients with PD demonstrated significant volumetric reductions in widespread white matter regions including fronto-limbic, thalamo-cortical and cerebellar pathways (p<0.05, FDR corrected). Furthermore, there was a significant negative relationship between right orbitofrontal gyrus (OFG) white matter volume and the severity of patients' clinical symptoms, as assessed with the PDSS. A significant positive relationship was also observed between patients' right OFG volumes and their scores on the GAF. Our results suggest that volumetric reductions in widespread white matter regions may play an important role in the pathology of PD. In particular, our results suggest that structural white matter abnormalities in the right OFG may contribute to the social, personal and occupational dysfunction typically experienced by patients with PD.

## Introduction

Multiple brain regions are thought to be affected in panic disorder (PD), including the limbic, frontal, and subcortical regions of the brain, as well as the brain stem [1]–[3]. Among these, the amygdala plays a crucial role in the development of PD symptoms.

Gorman et al (2000) have presented a neuroanatomical hypothesis of PD based on previous researches about human panic attacks and preclinical fear conditioning. Their hypothesis postulates that neurocognitive disturbances relating to viscerosensory information in cerebral regions (e.g., medial frontal region, cingulate gyrus, insula, thalamus, and hippocampus) could hyperactivate the amygdala [1]. More precisely, viscerosensory information is transferred to amygdala through two pathways. One pathway is so called “downstream” that includes solitary nucleus of medulla, parabrachial nucleus, and thalamus. Another pathway, “upstream”, is from primary viscerosensory cortex (insula) or via cortico-thalamic pathway to medial prefrontal cortex and cingulate gyrus having function of high-level neurocognitive and sensory information processing. Hyperactivation of the amygdala can, in turn, abnormally activate efferent target brain regions (e.g., hypothalamus, midbrain, and pons), leading to PD symptoms. Furthermore, Dresler et al. (2013) have suggested that Gorman's hypothesis depends on animal studies largely so that it might not sufficiently explain the neuropathology of PD. They have described that pathophysiological model of PD might be more intricate and more widely considering brain regions not only amygdala but also other brain regions such as insula and anterior cingulate gyrus (ACG). Anatomical model of PD was also expanded by other researchers. For example, Lai et al. (2012) have suggested that the orbitofrontal gyrus (OFG), inferior frontal gyrus, and superior temporal gyrus (STG) construct a sensory integration network which influences PD symptoms [2]. As described above, it has been suggested that pathophysiology of PD is related with abnormality of certain ‘network’ rather than that of a local brain region.

Previous neuroimaging studies have revealed structural and functional abnormalities in the gray matter regions of patients with PD compared with healthy control subjects (HCs) (structural: [2], [4]–[6], functional: [7]–[9]). Magnetic resonance imaging (MRI) studies have demonstrated gray matter volume abnormalities in these local brain regions in patients with PD. Specifically, significant volume reductions were found in limbic regions (amygdala [10] and insula [5]), frontal regions (OFG [6], ACG [4], and medial superior frontal gyrus [5]), thalamus [5], caudate nucleus [11], STG [2], [5], [12], and cerebellum [5], and significant increases in volume were found in the midbrain [13] and pons [14]. To the best of our knowledge, however, no study has reported on volume changes in white matter pathways connecting these gray matter regions in patients with PD.

PD is characterized by anxiety and viscerosensory disturbance [15]. Previous epidemiological studies have reported that patients with PD exhibit social dysfunction, and that this is related to symptom severity [16]. Social dysfunction is also reportedly associated with a lower quality of life [17], [18] and higher rate of suicide [18], [19] in patients with PD relative to healthy individuals. There is, thus, a need to examine the neuropathology underlying social dysfunction in these patients. Among the white matter regions, the OFG stood out as a strong candidate because it has been implicated in anxiety regulation and sensory integration [20], as well as social function [21]. Moreover, the OFG is involved in the prioritization of solutions to conflicts, a major concern among patients with PD [22]. The OFG also has extensive anatomical connections with the amygdala and ACG [21], [23], [24], both of which are involved in emotional and social functions [25]–[29] and thought to play crucial roles in the pathophysiology of PD. Gray matter volume reductions have been reported in these three brain regions in patients with PD [4], [6], [10].

In the current voxel-based morphometry (VBM) study, white matter volume abnormalities and their relationships with clinical symptoms and social function were investigated in patients with PD. Given previous reports of gray matter volume changes in such patients, we speculated that volume abnormalities would be observed in multiple white matter regions, including fronto-limbic regions (OFG and cingulum), thalamo-cortical pathways, insula, and cerebellum. We also hypothesized that white matter volume abnormalities in the OFG would be associated with symptom severity and social dysfunction in patients with PD.

## Materials and Methods

### Subjects

Subjects were 40 patients with PD (27 women and 13 men; “PD group”) and 40 HCs (27 women and 13 men; “HC group”). Patients were recruited from inpatient and outpatient units of Yokohama City University Hospital. HCs were recruited from the community and hospital staff. All participants met the following criteria: (1) right-handed and (2) no history of epilepsy, head trauma with loss of consciousness, neurological disorders, and/or substance abuse. Diagnosis was based on the Structured Clinical Interview for *DSM-IV* Axis I Disorders (SCID-I) [30]. This study included six patients with a past history of major depressive disorder, which is often associated with PD [31], and one patient with past history of anorexia nervosa who was within the normal body weight range when she participated in this study. Patients with current or past history of other psychiatric disorders were excluded from this study. Thirty-eight patients with PD and 37 HCs had participated in our previous study [13].

Patients had been receiving selective serotonin reuptake inhibitors (SSRIs) alone (*n* = 3); benzodiazepines alone (*n* = 7); SSRIs and benzodiazepines (*n* = 22); SSRIs and atypical antipsychotics (n = 1); serotonin-norepinephrine reuptake inhibitors (SNRIs) alone (n = 1); SNRIs and benzodiazepines (*n* = 2); SSRIs, SNRIs, and benzodiazepines (*n* = 1); tricyclic antidepressants and benzodiazepines (*n* = 2); and tetracyclic antidepressants, SSRIs, and benzodiazepines (*n* = 1).

HCs were confirmed to have no Axis I disorders with the SCID-NP (Non-patient Edition) [30] and the Mini-International Neuropsychiatric Interview [32], and no history of Axis I disorders in their first-degree relatives per self-report. None of the HCs had received psychiatric treatment.

The socio-economic status (SES) of all subjects and their parents was assessed with the Hollingshead Two-Factor Index [33]. Severity of illness and general level of functioning were evaluated with the Panic Disorder Severity Scale (PDSS) [34] and Global Assessment of Functioning (GAF).

This study was approved by the Medical Research Ethics Committee of Yokohama City University. After providing a complete description of the study, we obtained written informed consent from all participants.

### MRI processing

MR images were acquired with a 1.5-T Magnetom Symphony system (Siemens Medical System, Erlangen, Germany) at Yokohama City University Hospital. A series of 128 contiguous T1-weighted slices in sagittal images was acquired with a Turbo FLASH sequence with the following parameters: echo time  = 3.93 ms, repetition time  = 1960 ms, inversion time  = 1100 ms, flip angle  = 15, field of view  = 24 cm, matrix  = 256×256×128, and voxel dimensions  = 0.9375×0.9375×1.5 mm. Intracranial content volumes were calculated with a MATLAB function.

### Voxel-Based Morphometry

VBM was performed with the Diffeomorhphic Anatomical Registration Through Exponentiated Lie algebra (DARTEL) [35] tool in Statistical Parametric Mapping (SPM) 5 [36]. Baseline T1-weighted images were realigned so that the anterior and posterior commissure (AC-PC) line was horizontal and the midsagittal plane was vertical. The images were then segmented into probability maps of gray and white matter and cerebrospinal fluid by the unified segmentation approach in SPM5 [37]. These gray and white matter probability maps were rigid body aligned (3 rotations and 3 translations) to Montreal Neurological Institute (MNI) space and resampled into 1.5 mm isotropic voxels. Next, a gray matter template image was created by non-linearly registering all resampled gray and white matter probability maps using DARTEL. The gray matter maps of each subject were then spatially non-linearly normalized to the template. The white matter maps were registered to the template space using the same transformation parameters, followed by Jacobian modulation. In order to bring the final analysis into standard MNI space, the template was registered to the MNI space. All individual white matter maps residing in the template space were co-registered to MNI using the same affine transformation. Finally, these images were smoothed with an 8 mm full width at half maximum (FWHM) Gaussian kernel.

### Statistical analysis

The framework of the general linear model was used to estimate group differences in regional white matter volumes using a two-sample t-test. Intracranial content volume and sex were included as covariates. The resulting set of voxel values for each contrast constituted a statistical parametric map of the t statistic (SPM (t)). SPM (*t*) maps are displayed at an uncorrected threshold of p<.001, with an extent threshold of 40 voxels for graphical reporting. In the text and tables, results that survived a correction at False Discovery Rate (FDR)-corrected p<.05 for the volumes searched are discussed [36], [38].

### Volume-Symptom Relationships

Regional volumes were calculated as described by Asami [36] and Whitford [39]. First, regions for which the VBM analysis showed significant white matter volume reductions in patients with PD relative to HCs were extracted as a gray-scale image using the ‘save’ option on the SPM5 graphic user interface. Following this, the gray-scale image was parcellated into each cluster using 3D-Slicer (http://www.slicer.org/), and binary images for each cluster were created. These binary images were convolved with all pre-processed white matter images, and volumes of each cluster were calculated by summing the constituent smoothed modulated values. Relative volumes of each cluster were then calculated.

To evaluate associations between structural changes and clinical symptoms and social function in patients with PD, Spearman's correlation analyses were conducted between regional white matter volumes and scores of the PDSS and GAF. Relationships between medication dosage and structural changes were also evaluated in the patients with PD [40]. The significance threshold was set at p<.01 (two tailed) to correct for multiple comparisons. Statistical analyses were performed using PASW Statistics version 18 for Windows (SPSS Inc.).

## Results

### Clinical features

Demographic information for each group is summarized in **Table 1**. There were no significant differences in age, sex, and parental SES between the two groups. The self SES was lower, but not significant, in patients with PD compared to HCs.

**Table 1:** Demographic and clinical characteristics of the subjects.

	patients with PD			control subjects			Mann-Whitney U test^a^
Characteristic or test	N	Mean	SD	N	Mean	SD	p
Sex (M/F)	13/27			13/27			
Age (years)	40	39.2	10.5	40	37.4	10.3	.434
Self SES^b^	40	2.5	0.9	40	2.1	0.9	.069
Parental SES^b^	40	2.6	0.8	40	2.4	0.8	.291
GAF	40	64.7	11.3				
PDSS	39	10.6	5.8				
Panic attack frequency	39	1.05	0.92				
Distress during panic attacks	39	1.90	1.25				
Severity of anticipatory anxiety	39	1.51	0.85				
Agoraphobic fear/avoidance	39	1.56	1.07				
Panic-related sensation fear/avoidance	39	1.49	1.12				
Impairment/interference in work functioning due to panic disorder	39	1.51	1.30				
Impairment/interference in social functioning due to panic disorder	39	1.54	1.02				
Age of first medication (years)	40	35.3	10.2				
Duration of illness (years)	40	5.2	6.4				

Abbreviations: PD, panic disorder; SES, socio-economic status; GAF, global assessment of functioning; PDSS, panic disorder severity scale.

a Mann-Whitney U tests were performed between the two groups for age, self SES, parent SES.

b Higher scores mean lower socioeconomic status, based on the Hollingshead two factor index of socioeconomic status.

### Voxel-Based Morphometry

**Figure 1:**
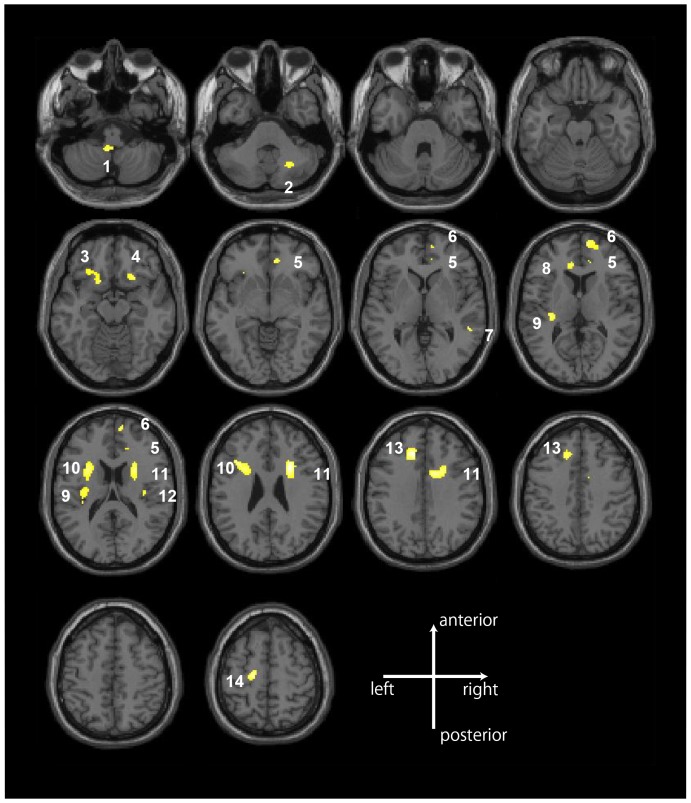
White matter volume reductions in the patients with panic disorder compared with the healthy control subjects. The patients with panic disorder had significant volume reductions in 14 distinct white matter regions (clusters) compared with the healthy control subjects (p<.05, FDR-corrected). The 14 white matter regions included fronto-limbic regions (bilateral OFG, right superior frontal gyrus, left precentral gyrus, bilateral cingulum, and insula), thalamo-cortical pathways (anterior and superior corona radiata), right superior and middle temporal gyrus, and the cerebellum.

The VBM analysis revealed that the PD group had significant volume reductions in 14 distinct white matter regions (clusters) compared with the HC group (p<.05, FDR-corrected) (**Figure 1** and **Table 2**). These 14 clusters were anatomically identified using Mori's white matter atlas [41], which was previously registered to the MNI space. For non-named white regions, anatomical identification was conducted using information from related gray matter regions.

**Table 2:** White matter volume reductions in the patients with panic disorder compared with the healthy control subjects.

cluster number	Anatomical location	Number of voxels	MNI coordinates			T value	Z value	Uncorrected p	FDR-corrected p
			x	y	z				
1	left inferior cerebellar peduncle	68	−6	−46	−48	3.54	3.40	<.001	.037
2	right cerebellum	56	26	−64	−36	3.55	3.41	<.001	.037
3	adjacent to left OFG	125	−16	16	−12	3.50	3.36	<.001	.037
	left anterior corona radiata		−28	24	−10	3.47	3.33	<.001	.037
	left inferior fronto-occipital fasciculus								
4	adjacent to right OFG	42	22	20	−14	3.52	3.38	<.001	.037
5	adjacent to right ACG	53	10	42	−4	3.72	3.55	<.001	.037
			10	42	6	3.31	3.19	.001	.037
			14	38	12	3.28	3.16	.001	.037
6	adjacent to right superior frontal gyrus	118	10	58	8	3.84	3.66	<.001	.037
			10	60	18	3.65	3.49	<.001	.037
			22	56	12	3.47	3.34	<.001	.037
7	adjacent to right superior and middle temporal gyrus	42	54	−38	0	4.09	3.88	<.001	.037
			58	−48	−2	3.53	3.38	<.001	.037
8	left cingulum	52	−8	36	8	3.53	3.38	<.001	.037
9	adjacent to left insula	182	−32	−24	8	3.62	3.47	<.001	.037
			−34	−18	18	3.43	3.30	<.001	.037
			−34	−32	16	3.27	3.15	.001	.037
10	left anterior and superior corona radiata	306	−36	16	28	4.01	3.81	<.001	.037
			−28	10	22	3.88	3.70	<.001	.037
11	right superior corona radiata	592	26	12	26	4.18	3.95	<.001	.037
	right cigulum		14	4	36	3.89	3.70	<.001	.037
	right body of corpus callosum								
12	adjacent to right insula	40	36	−18	16	3.52	3.38	<.001	.037
13	adjacent to left ACG	229	−12	28	36	4.21	3.98	<.001	.037
14	adjacent to left precentral gyrus	63	−18	−18	58	3.83	3.65	<.001	.037

Abbreviations: MNI, montreal neurological institute; ACG, anterior cingulate gyrus; OFG, orbitofrontal gyrus; FDR, false discovery rate.

The 14 white matter regions included fronto-limbic regions (bilateral OFG, right superior frontal gyrus, left precentral gyrus, bilateral cingulum, and insula), thalamo-cortical pathways (anterior and superior corona radiata), right superior and middle temporal gyrus, and the cerebellum (**Figure 1**, **Table 2** **, and Supplemental Material**). The PD group showed no volume increases compared to the HC group.

### Correlation analysis

**Figure 2:**
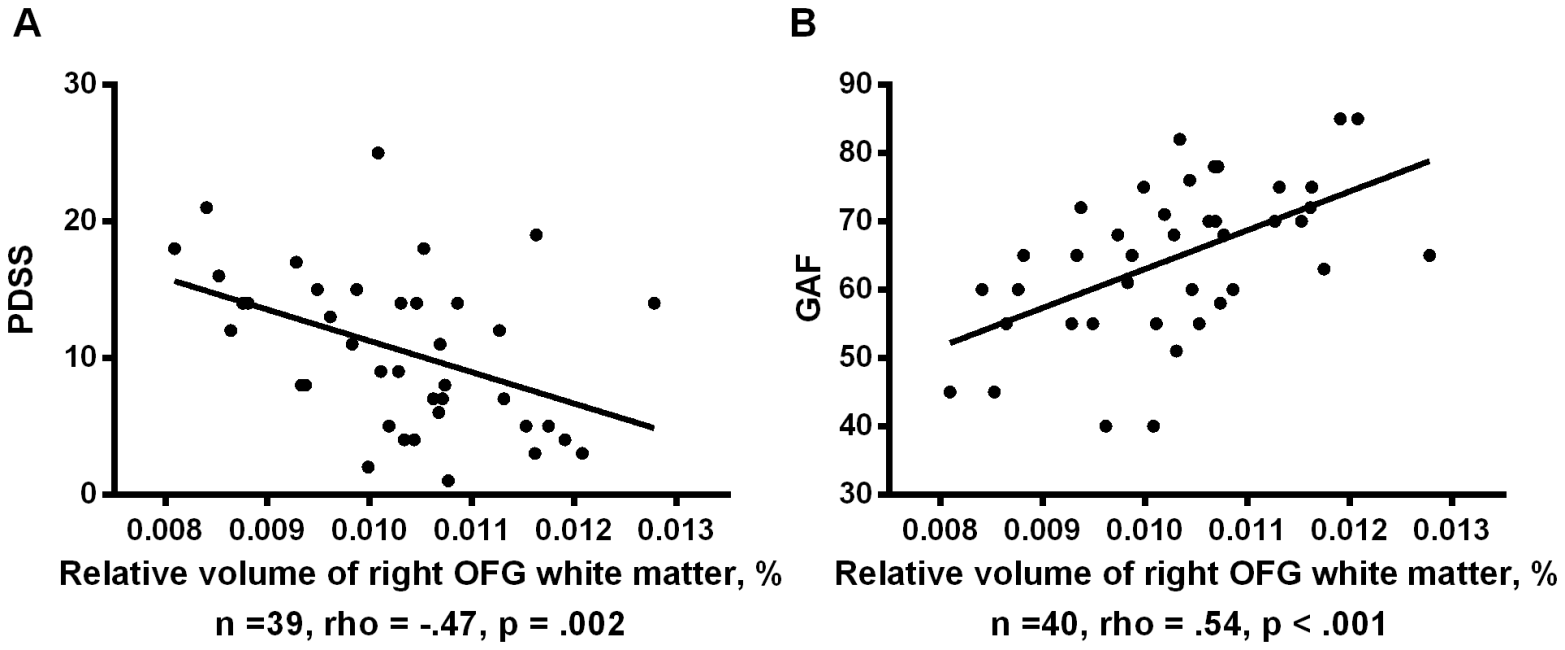
Relationships between white matter volumes of the right OFG and scores of PDSS and GAF in the patients with panic disorder. The white matter volume of the cluster in the right OFG was significantly negatively related to total PDSS scores in the patients with panic disorder (rho = −.47, p = .002) (A). The white matter volume of the cluster was also significantly positively related with GAF scores in the patients with panic disorder (rho = .54, p<.001) (B).

The white matter volume of Cluster 4 in the right OFG was significantly negatively related to total PDSS scores in the PD group (rho = −.47, p = .002, **Figure 2A**). Additional correlation analyses were conducted to evaluate relationships between Cluster 4 volume and scores of each PDSS item with the cut-off p value set at .01. Cluster 4 volumes were significantly negatively related with the following PDSS items: “Panic-related sensation fear/avoidance” (rho = −.44, p = .005), “Impairment/interference in work functioning due to panic disorder” (rho = −.46, p = .003), and “Impairment/interference in social functioning due to panic disorder” (rho = −.45, p = .004). The white matter volume of Cluster 4 was significantly positively related with GAF scores in the PD group (rho = .54, p<.001, **Figure 2B**). No other cluster showed significant relationships with scores of the PDSS or GAF in the PD group. In terms of associations between medication dosage and structural changes, no white matter regions had significant relationships with antidepressant dosages in the patients with PD. White matter volume of Cluster 8 (left cingulum) demonstrated a negative relationships with benzodiazepine dosages (rho = −.44, p = .005). Other clusters showed no relationship with the dosage of benzodiazepines.

## Discussion

In this study, significant volume reductions were found in multiple white matter regions in patients with PD compared with HCs. To the best of our knowledge, this is the first study reporting abnormalities in local white matter volumes in patients with PD. These regions, as we speculated, included white matter of fronto-limbic regions (bilateral OFG, cingulum, insula, right superior frontal gyrus, and left precentral gyrus), thalamo-cortical pathways (anterior and superior corona radiata), and the cerebellum. Our VBM analyses also showed white matter volume reductions in the superior and middle temporal gyrus.

It has been suggested that pathophysiology of PD is associated with fear network. Sensory information is thought to be transferred to the amygdala through the two pathways, the “downstream” including solitary nucleus of medulla, parabrachial nucleus, and thalamus, and the “upstream” including insula, medial prefrontal cortex and ACG [1]. Furthermore, expanding this conventional hypothesis, insula, ACG, and STG are also thought to play important roles in fear network [2], [42]. These have led to the notion that neurocognitive disturbances in these gray matter regions could lead to a misinterpretation of sensory information, further leading to inappropriate hyperactivation of the amygdala and development of PD symptoms. Indeed, previous studies have reported volume reductions in these gray matter regions in patients with PD [2], [4]–[6], [10]. In the current VBM study, volume reductions were observed in white matter connecting these gray matter regions which constructed fear network. Structural abnormalities in these white matter pathways might also contribute to the pathology of PD by provoking miscommunication between certain gray matter regions, which could lead to misinterpretation of sensory information, resulting in abnormal activation of the fear network.

Further to our previous study demonstrating gray matter volume reductions [5], the present study also revealed white matter volume reductions in the cerebellum in patients with PD. The cerebellum is intimately related to anxiety and PD [3]. Recent functional neuroimaging studies involving patients with PD have reported the possible inclusion of the cerebellum in the “fear circuit” [9], and have shown decreased activation of the cerebellum after cognitive-behavioral therapy in patients with PD [43]. Other studies have reported an altered functional connectivity between the amygdala and cerebellum in patients with other anxiety disorders, such as generalized anxiety disorder [44], [45]. Thus, structural deficits in cerebellar white matter observed in the present study may contribute, at least in part, to functional abnormalities associated with symptom appearance in patients with PD.

Given previous studies showing that the severity of mental illness increased disability [16] and that patients with PD had lower social functioning and quality of life relative to HCs [17], [46], [47], we believed it important to identify white matter regions associated with symptom severity and social function. Consistent with an association with social function, self SES scores of our patients were lower than those of matched HCs. Evidence also exists regarding interactions between anxiety and social function, in which anxiety can induce social dysfunction [48] and worsening social dysfunction can increase risk of panic disorder [49]. Thus, understanding the pathology of symptom severity and social dysfunction in patients with PD is important, and this led us to focus on the OFG, which is believed to be involved in both emotional and social function.

In our correlation analyses, right OFG white matter volume (Cluster 4) showed strong negative relationships with total PDSS scores and PDSS items related to symptom severity and social function. Right OFG white matter volume was also positively associated with GAF scores

In terms of the relationship between white matter volumes of the right OFG and the symptom severity, the right OFG white matter volume showed a negative association with the PDSS item “Panic-related sensation fear/avoidance.” The OFG regulates fear and anxiety, both of which are principle components of PD symptoms. Consistent with this, an animal study reported that introducing an anterior OFG lesion in marmosets led to stronger fear responses, suggesting that the anterior OFG attenuates fear and anxiety [50]. In humans, functional neuroimaging studies have shown increased activity in the OFG during fear conditioning [51], and when autonomic response in fear conditioning was attenuated, activity in the OFG decreased in parallel [52]. OFG activation is also related to anticipatory anxiety, hyperventilation, and fear of somatic symptoms, which are all symptoms of PD, in healthy individuals [53], [54]. Moreover, several studies have also demonstrated relationships between abnormal OFG activation and fear and anxiety in patients with PD [8], [55]. For example, the OFG and other regions, such as anterior and posterior cingulate cortices, were activated when patients with PD were exposed to their anxiety-provoking episodes [55]. In a study involving doxapram, a respiratory stimulant which induces anxiety and panic attacks, cerebral blood flow in the OFG was negatively correlated with anxiety scores on the Acute Panic Inventory and the 10-point Anxiety Scale [8]. Thus, OFG white matter volume reductions may contribute to greater symptom severity in patients with PD.

The OFG is known to make extensive connections with the amygdala and medial prefrontal cortex including the ACG [21], [23], [56]. A meta-analysis of functional imaging studies revealed that the medial OFG is connected with these two regions [57]. Previous animal and human studies have established a role for the amygdala and ACG in anxiety and emotional regulation [26], [27], [53], [58], [59]. These regions are, thus, thought to play an important role in PD pathology [1]. Indeed, previous studies have shown abnormal activity in these regions, and have revealed their relationships with anxiety and fear severity in patients with PD [9], [43], [55], [60]. Our previous results demonstrating reduced gray matter volume in these regions in patients with PD [4], [5], [10] suggest the possibility that white matter volume reduction in the right OFG may also play an important role in the pathology of anxiety and fear in PD. This is likely relevant not only in fear-related gray matter regions with white matter volume reduction (i.e., the OFG itself), but also white matter pathways that connect fear-related gray matter regions (the OFG, amygdala, and ACG).

About the association between white matter volumes of the right OFG and the social function, the right OFG white matter volume showed negative relationships with the PDSS items “Impairment/interference in social functioning due to panic disorder,” which evaluates interpersonal functioning, and “Impairment/interference in working functioning due to panic disorder.” The OFG and medial prefrontal cortex integrate various stimuli, such as somatosensory information, and produce outcomes [21]. For example, the OFG is thought to integrate signals encoding stimulus value in collaboration with the medial prefrontal cortex, including the ACG, from other brain regions in order to calculate net values of reward and action [61], and regulate the response of limbic regions and behavior [62]. Thus, the OFG integrates approach- and avoidance-valuations [22]. White matter deficits in the OFG could lead to misintegration of approach-avoidance valuation, resulting in decreased interpersonal function in patients with PD.

The relationship between the OFG and general social and working functions have been previously reported. For instance, a subject with bulk damage to the OFG began to experience social dysfunction, such as being fired from jobs and relying on family support [63], [64]. In a different study, a subject, after surgery for meningioma in the OFG, did not exhibit reduced IQ, but lost his job and family [65]. It has also been reported that deficits in the OFG reduce the ability to identify errors in social situations [66], leading to an impaired theory of mind [67], [68]. Thus, structural deficits in OFG white matter may underlie social and working dysfunction in patients with PD.

Previous studies have revealed that the amygdala and medial prefrontal cortex, including the ACG, which are anatomically and functionally connected with the OFG, are associated with interpersonal, social, and working functioning. The amygdala is involved in trustworthiness judgment and theory of mind, as reported in studies of subjects with damaged amygdala [69]–[72] and functional MRI studies of normal individuals [73], [74]. Other studies have reported an association between the medial prefrontal cortex and social judgment [75], [76] and social emotion, such as embarrassment and guilt [77]. The ACG is associated with social stimuli, such as cooperation [78], exclusion [28], and rejection [29]. Schweizer et al. (2013) noted that the emotional control capacity in the ACG is associated with working and interpersonal success [79]. These findings related to the structural-functional relationships between the OFG, amygdala, and ACG, and interpersonal, social, and working functioning, are consistent with our findings that volume reductions in white matter pathways connecting these three regions provoke social and work dysfunction in patients with PD.

Finally, we found a positive relationship between right OFG white matter volumes and GAF scores, which measures psychological, social, and occupational functioning [15], in patients with PD. This finding was in line with the relationships described above, in which lower white matter volume in the right OFG is associated with heightened fear/avoidance and worse social and working functioning.

The study has some limitations worth noting. First, this study only evaluated group differences in white matter volumes. However, a diffusion tensor imaging study with tractography would be needed to evaluate structural abnormalities in white matter pathways [80]. Second, gender differences have been observed in the epidemiology and symptoms of PD, as well as regional gray matter volume abnormalities [5]. Thus, gender differences in white matter volumes will need to be assessed in a large population in the future. Thirdly, our correlation analyses have a multiple comparison issue. Our results are needed to be confirmed in future studies with large population. Finally, all of our patients received medication. Although our results showed or not showed associations between medication dosage and regional white matter volumes, medication effects on white matter volumes were still unclear. Further studies with drug naïve patients will be needed to evaluate pure effect of PD on white matter volume changes in future.

## Conclusions

In conclusion, our study demonstrated white matter volume reductions in multiple brain regions, including fronto-limbic regions (e.g., OFG, ACG, and insula), thalamo-cortical pathways (e.g., corona radiata), temporal regions, and the cerebellum. Among these regions, white matter volume in the right OFG was associated with PDSS total scores and the PDSS items “Panic-related sensation fear/avoidance” and “Impairment/interference in social and working functioning due to panic disorder.” OFG white matter volumes were also related with GAF scores, which evaluate psychological, social, and occupational functioning. These results suggest that in addition to gray matter volume abnormalities in frontal, limbic, and temporal regions, and the cerebellum, reduced volume of white matter connecting these regions likely plays important roles in PD pathology. Moreover, structural abnormalities in right OFG white matter contribute to fear/avoidance, as well as social and working dysfunction in patients with PD.

## Supporting Information

Figure S1 **White matter volume reductions in the patients with panic disorder compared with the healthy control subjects.** Glass brain pictures show white matter volume reductions in the 40 patients with panic disorder compared with the 40 healthy control subjects. Uncorrected p<.001 with an extent threshold of 40 voxels was used for graphical reporting, and all the regions shown as volume reductions also satisfied the False Discovery Rate criterion with a corrected P<.05. Abbreviations: A, anterior; P, posterior; R, right; L, left.
(TIF)
