# Supplementary figures and images for: Multiple White Matter Volume Reductions in Patients with Panic Disorder: Relationships between Orbitofrontal Gyrus Volume and Symptom Severity and Social Dysfunction
Source: PLoS One. 2014 Mar 24;9(3):e92862. doi: 10.1371/journal.pone.0092862 (PMC3963974; doi:10.1371/journal.pone.0092862)

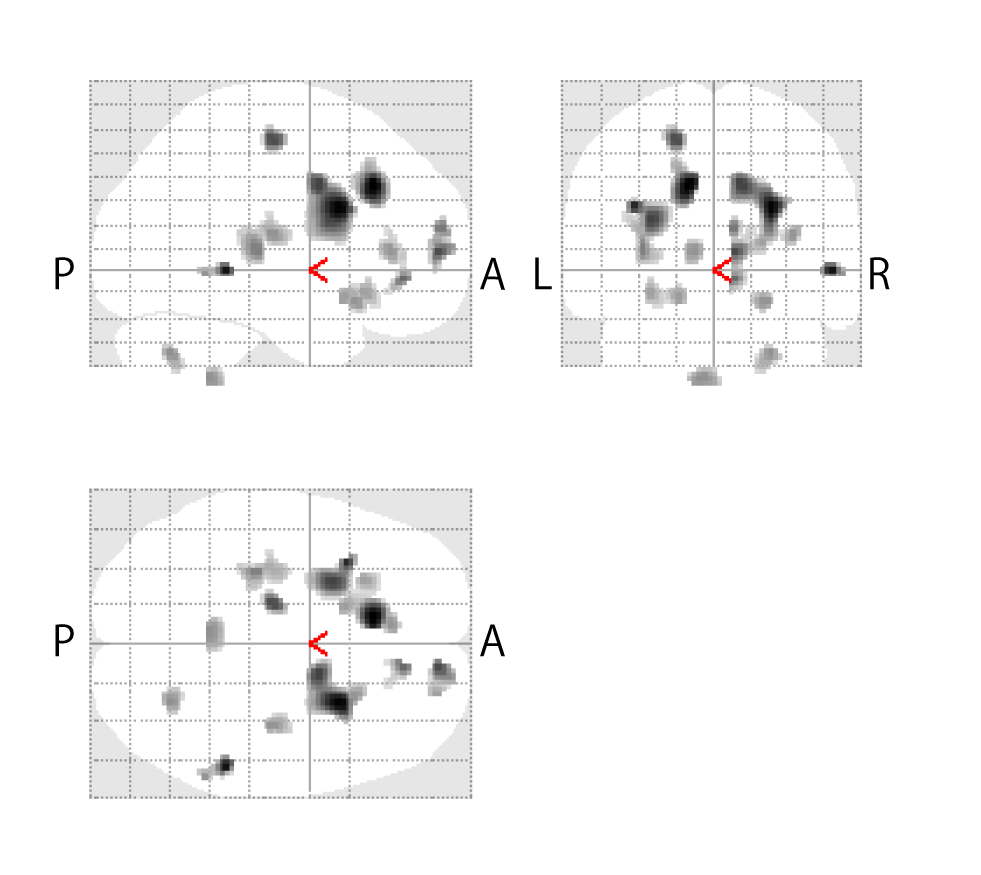

Supplement: Figure S1 — White matter volume reductions in the patients with panic disorder compared with the healthy control subjects. Glass brain pictures show white matter volume reductions in the 40 patients with panic disorder compared with the 40 healthy control subjects. Uncorrected p<.001 with an extent threshold of 40 voxels was used for graphical reporting, and all the regions shown as volume reductions also satisfied the False Discovery Rate criterion with a corrected P<.05. Abbreviations: A, anterior; P, posterior; R, right; L, left. (TIF) [file pone.0092862.s001.tif]
